# Molecular Photogearing for Controlling Rotary Motion at the Nanoscale

**DOI:** 10.1002/cplu.202500742

**Published:** 2026-04-05

**Authors:** Enrique M. Arpa, Bo Durbeej

**Affiliations:** ^1^ Institute of Organic Chemistry RWTH Aachen University Aachen Germany; ^2^ Division of Theoretical Chemistry IFM Linköping University Linköping Sweden

**Keywords:** isomerization, molecular devices, molecular dynamics, photochemistry, steric hindrance

## Abstract

Means to control rotary motion at the nanoscale are central to the design and operation of artificial molecular machines powered by such motion. For this task, it is natural to consider molecular gears, which are characterized by their ability to perform coupled rotations around two (or more) chemical bonds. However, most such gears rely on passive, thermal activation, which makes them sensitive to Brownian motion. In this concept, following a brief review of the historic development of molecular gears, we highlight some recent experimental and computational results that have helped show how this problem can now be addressed by means of the type of molecular photogearing achieved when the double‐bond rotary motion produced by a light‐activated molecular motor is transmitted through space onto a single‐bond axis. Furthermore, we discuss the formidable challenge to maintain a preferred direction of rotation during this transmission, which is critical for performing mechanical work. Finally, we point out some research directions suitable for maximizing the future usefulness of molecular gears and photogears.

## Graphical abstract

Molecular gears capable of controlling and transmitting rotary motion at the nanoscale are indispensable for the construction of artificial molecular machines. In this concept, we discuss how molecular photogears featuring a propeller‐shaped motif attached to a light‐driven molecular motor offer new possibilities to perform these tasks with better control of the direction of rotation.

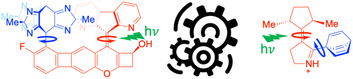

## 1 Introduction

Gears are at the heart of nearly any manmade machinery devised in the modern era. Through their ability to synchronize and transmit rotary motion, gears are critical for separating machines into motor and actuator parts responsible for generating the rotary motion and using it to carry out the desired function, respectively. Interestingly, gears are also found in Nature, such as in the legs of some planthoppers, who use them to coordinate their jumps [1].

The continuous miniaturization of technology has taken the design of ever‐smaller machines to unprecedented scales [2, 3]. Indeed, since the beginning of the 21st century, a plethora of artificial molecular machines carrying out a wide variety of functions has been manufactured [4, 5, 6, 7, 8, 9]. Still, among those machines featuring a motor, a common limitation is that the motor and actuator parts are fused in a single entity. Accordingly, if a desired function requires multiple actuators to perform mechanical work, then each of them needs to be supported by their own motor. However, in this scenario, optimal functionality typically demands that all actuators are activated simultaneously, which is not necessarily so easy. Thus, a natural goal is to rather devise artificial molecular machines where all actuators are powered by a single motor. For this, one needs molecular gears capable of transmitting rotary motion at the nanoscale in a controlled manner [10, 11].

In this article, following a brief review of the field of molecular gears starting from its inception almost half a century ago [12, 13], we outline recent advances in the field made by invoking the evolving concept of molecular photogearing [14, 15, 16, 17]. In particular, we describe ways to exploit this concept discovered in our own computationally oriented research in the field [16, 17]. Given that the previous experimental work upon which this research is mostly based pertains to intramolecular gearing in solution [14, 15], this article then maintains the same focus, notwithstanding recent advances in molecular gearing in the solid state and on surfaces [18, 19].

## 2 Transmission of Rotary Motion at the Molecular Level

**FIGURE 1:**
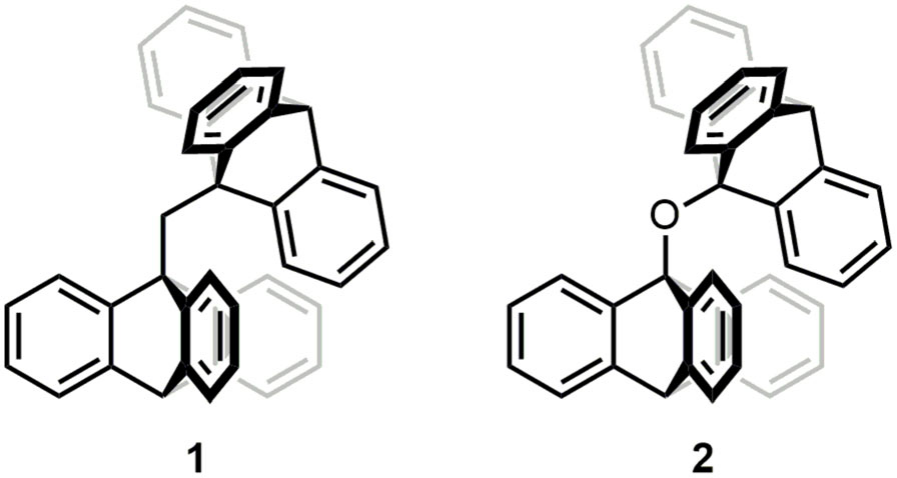
Bis(9‐triptycyl)methane (left) and bis(9‐triptycyl)ether (right) molecular gears.

The first molecular gears were developed independently by the groups of Iwamura and Mislow in 1980 [12, 13, 20]. At that time, it was known that, for molecules where two propeller‐shaped triptycyl groups are linked to a common center at their bridgehead positions, steric hindrance can render the rotational barriers with respect to these single‐bond linkages high enough to effectively prevent each group from undergoing independent rotation. Despite this, Kawada and Iwamura reported two compounds of this type (bis(9‐triptycyl)methane **1** and bis(9‐triptycyl)ether **2**, see Figure 1) where, quite unexpectedly, the triptycenes show fast *coupled* rotation even at low temperatures [12]. Indeed, tracking ^1^H‐NMR and ^13^C‐NMR spectral changes down to –94°C, it was inferred that the barrier for the coupled rotation is small, regardless of the fact that these compounds are sterically congested. Later that same year, Mislow and coworkers performed empirical force‐field calculations to demonstrate the existence of two very different transition states for the rotation of the triptycenes [13]. With a 1 kcal mol^–1^ barrier, the lower‐energy one was associated with true molecular gearing, as characterized by coupled, simultaneous rotation of the two triptycene cogwheels in a way akin to the disrotatory motion sustained by a “real” macroscopic gear—the clockwise rotation of one cogwheel is transmitted into a counterclockwise rotation of the adjacent cogwheel. Contrarily, with a considerable 20 kcal mol^–1^ barrier, the higher‐energy transition state was found to involve gear slippage, a process in which one of the cogwheels rotates in an arbitrary direction and the other stays still [13].

The term “molecular gear” seems to have been coined by Mislow and coworkers in 1981 [21], in a study following up on their aforementioned 1980 work [13]. Specifically, seeking conclusive experimental evidence for gearing, these authors chose to investigate a related bis(2,3‐dimethyl‐9‐triptycyl)methane compound where the C_3_ symmetry of the triptycenes is broken, which enabled the authors to record suitable NMR‐based evidence [21]. In 1983, the Iwamura group then made a further refinement in terminology through their introduction of “molecular bevel gear” [22]. In modern parlance [11], this means that the two rotation axes are intersecting, whereas they are parallel in a “molecular spur gear.”

**FIGURE 2:**
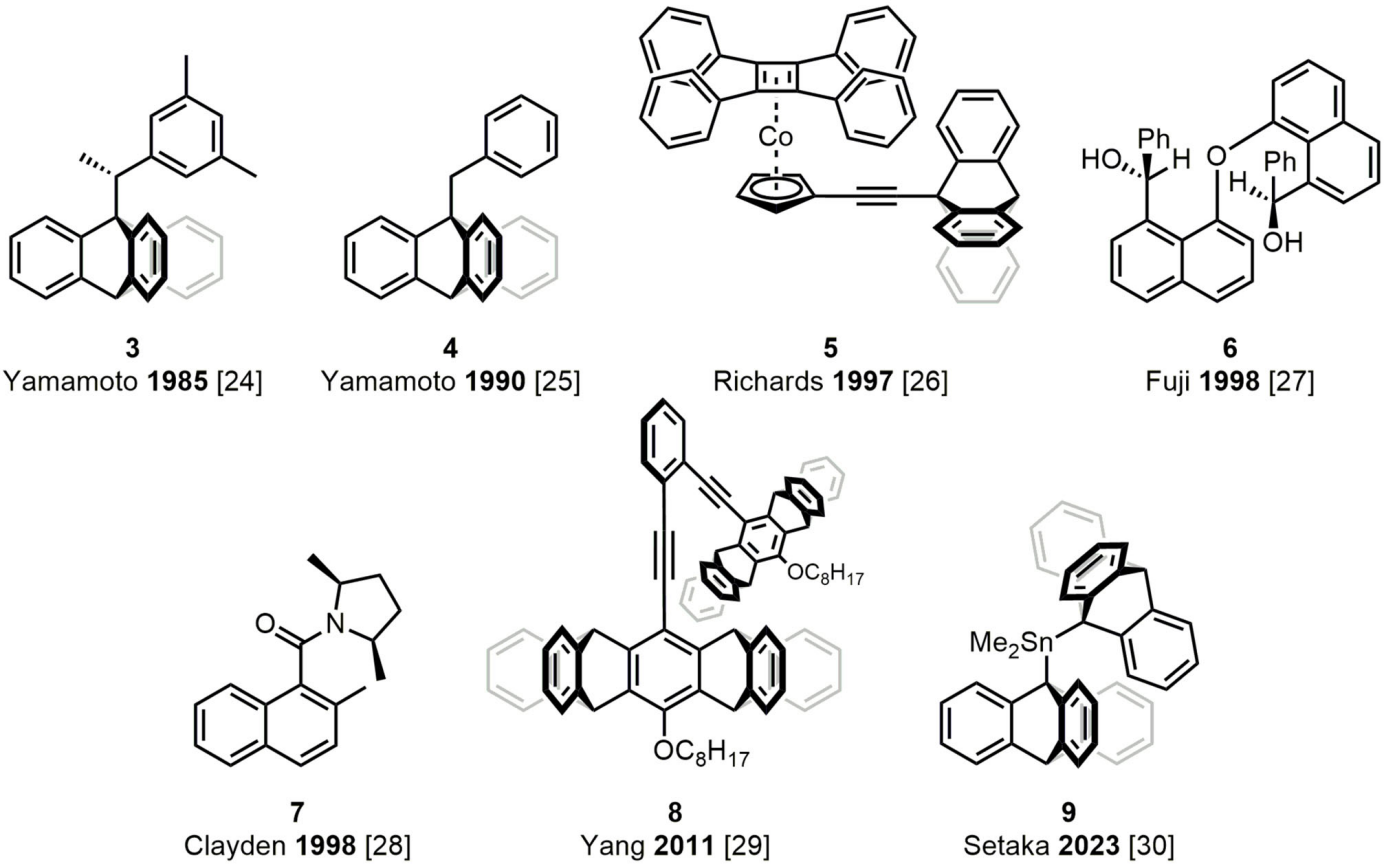
Examples of different molecular bevel gears and the years they appeared.

Using triptycenes as the two cogwheels in a molecular gear is advantageous not only in terms of synthetic feasibility [23] and chemical stability (because of aromaticity), but also in terms of the van der Waals volume (or steric hindrance) needed for gearing. In fact, in a gear composed of two triptycenes, their rotations are correlated in such a way that a 120° (C_3_ symmetry) clockwise rotation of one of them is necessarily coupled to a 120° counterclockwise rotation of the other. However, depending on the actual application, it may be desirable to break this symmetry, which can be done by replacing one or both of the triptycenes with other groups (see Figure 2). To this end, starting in 1985, Yamamoto reported bevel gears in which one of the triptycenes is replaced by a xylyl [24] (**3** in Figure 2) or an unsubstituted phenyl group [25] (**4**). In this way, a nonequal gearing ratio can be afforded, with the rotation of the aryl group exceeding that of the triptycene. Similarly, in 1997, Stevens and Richards substituted one of the triptycenes with a cobaltocene, which was connected to the remaining triptycene through an alkynyl linkage [26] (**5**). The resulting bevel gear was also found to afford a nonequal gearing ratio, but this time with the cobaltocene rotation lagging behind the triptycene rotation. This work was also the first to show that the phenomenon of molecular gearing is not exclusive to purely organic molecules, but can also be realized in organometallic systems.

As further illustrated in Figure 2, bevel gears containing no triptycene motif are also available. For example, while the triptycene‐based gears in Figure 1 include three cogs on each of the two wheels, in 1998 Fuji and Kinoshita reported the first gear featuring only two cogs on each wheel, as made possible by connecting two identical naphthalenes through an ether linkage [27] (**6**). Closely related to, but independent of, this work, Clayden and Pink instead prepared an unsymmetric gear where the rotation of a naphthalene around a CC bond is coupled to the rotation of an amide group around a CN bond [28] (**7**). This latter gear deserves a special mention in that, at room temperature, such rotations are not expected to be particularly facile in a rigid peptide‐bond environment. However, performing variable‐temperature NMR measurements to obtain relevant kinetic parameters at 33°C, the two rotations were actually predicted to proceed with a high degree of concertedness. Besides reducing the number of cogs on each wheel, progress has also been made in the opposite direction, such as by Yang and coworkers, who in 2011 put forth a pentiptycene‐based gear with four cogs on each of the two wheels [29] (**8**). Other researchers have generalized the basic core structure underlying Iwamura and Mislow's original gears [12, 13, 20] by showing that it is possible to replace the associated methylene or ether linkage by connectors based on other elements than C and O. Along this line, one may highlight the 2023 work by Setaka and coworkers, who used dimethyltin and diphenyltin connectors [30] (**9**).

**FIGURE 3:**
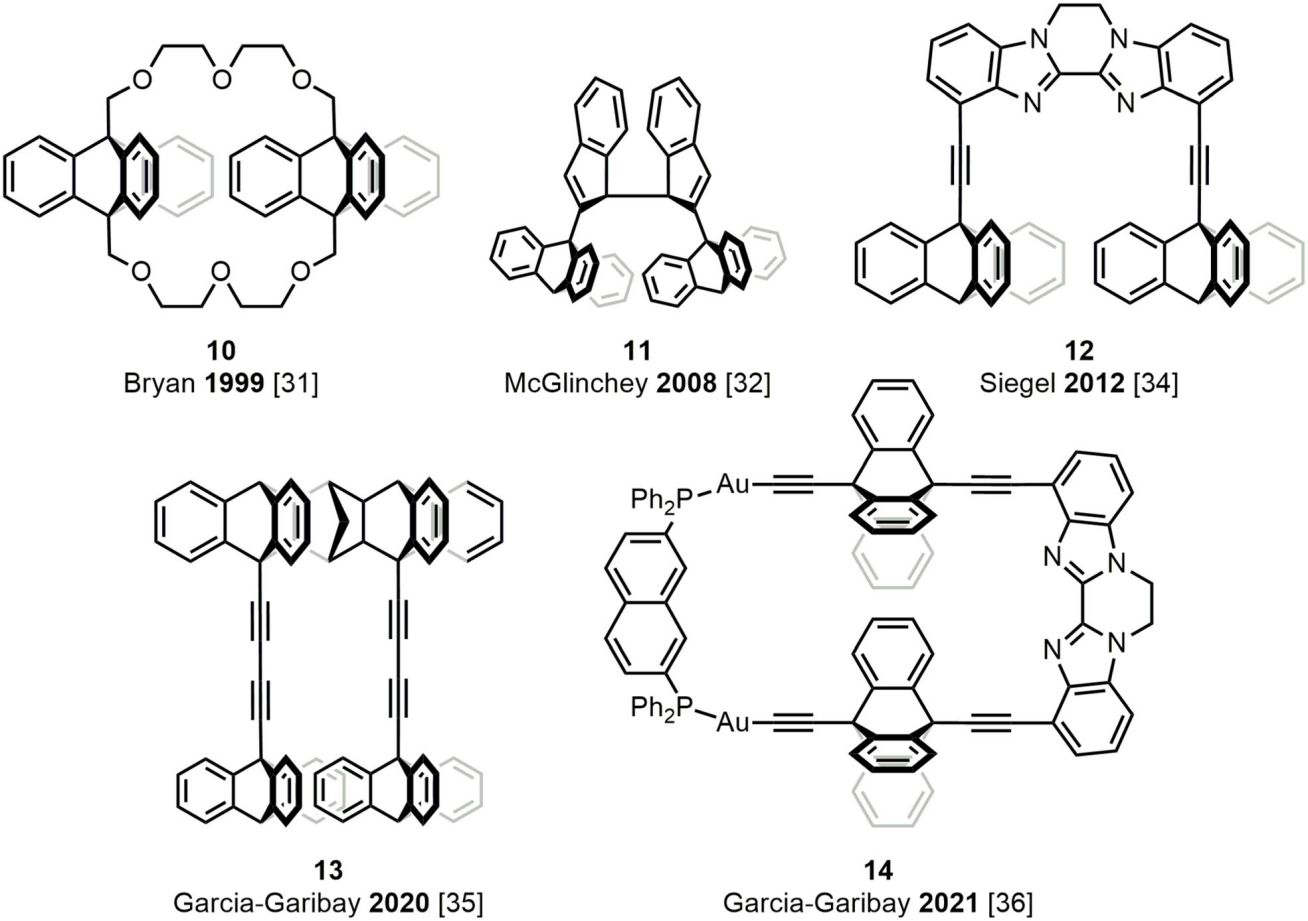
Examples of different molecular spur gears and the years they appeared.

All molecular gears described up to this point have their rotating wheels adopt a bevel‐type configuration, which simply reflects that it took almost 20 years after Iwamura and Mislow's groundbreaking work on bevel gears [12, 13, 20] before Bryan and coworkers could overcome the associated synthetic challenges and present the first spur gears in 1999 (see **10** in Figure 3) [31]. In order to allow for a spur‐type configuration (and provide some structural rigidity to maintain it), these researchers inserted two triptycenes at opposite ends of differently sized crown ethers, using both bridgehead positions of both triptycenes as connection points. Later, in 2008, McGlinchey and coworkers found a way to realize the same goal by connecting the triptycenes to a rigid bis‐indenyl core, but this time by engaging only one of the bridgehead positions of the triptycenes [32] (**11**). Despite this progress, the most critical parameter for the design of a spur gear is the distance between the rotation axes, as lucidly discussed by Siegel and coworkers in 2009 [33]. Specifically, in a spur gear, the two wheels are necessarily connected to different sites of a static (and ideally rigid) core structure, be it a bis‐indenyl in the aforementioned design by McGlinchey [32], or other structures in subsequent designs by Siegel and coworkers in 2012 [34] (**12**) and Garcia‐Garibay and coworkers in 2020 [35] (**13**) and 2021 [36] (**14**). Through this requirement, which does not apply to bevel gears, it becomes possible to control the distance between the rotation axes by modifying the size of the core structure. Naturally, in a macroscopic spur gear made of a rigid, solid material, this distance cannot be smaller than the sum of the radii of the two wheels. Molecular spur gears, on the other hand, are clearly more flexible, which means that this limitation is lifted. Nonetheless, Siegel determined that gearing (coupled rotation of the two wheels) is only efficient in a narrow range of interaxis distances [33]. Above this range, the wheels are no longer intermeshed and thus rotate independently, and below it, gear slippage (one wheel rotates and the other stays still) becomes the more favorable process. Subsequent research has then confirmed the importance of this distance for the development of spur gears [34, 35, 36], which nevertheless remains less advanced than that of bevel gears [11].

Regardless of whether their wheels have a bevel‐ or spur‐type orientation, all molecular gears described so far contain just two wheels. Hence, if one would like to transmit rotary motion over distances larger than the corresponding molecular dimensions, one must either increase the number of wheels or their sizes, or find a way to exploit “trains” of multiple gears where the rotation is successively transmitted from gear to gear (akin to how rotary motion is transmitted in many macroscopic machines). Along the first of these lines, following early studies by Mislow of coupled rotations of three aryl rings in molecular propellers [37], Kobayashi and coworkers have described a supramolecular gear with four Rh‐porphyrin wheels linked to a cavitand scaffold [38], thus illustrating the feasibility of gearing in a really large molecular assembly. Similarly, Shionoya and coworkers have reported a circularly arranged sextuple triptycene gear molecule [39]. Along the second line, several groups have worked toward attaching trains of spur gears to different surfaces, such as gears based on star‐shaped Ru complexes [40], hexaphenylbenzenes (attachable to Cu adatoms on the Pb(111) surface) [41], or pentaphenylcyclopentadienyl radicals (attachable directly to the Au(111) surface) [42].

## 3 Light‐Mediated Control of Rotary Motion

Despite the advances outlined in the previous section, the usage of these molecular gears in solution‐based nanomachines is challenged by the fact that their gearing is triggered through passive, thermal activation. Indeed, this working mode is not ideal for the construction of nanomachines that one would like to operate in a time‐controlled fashion, meaning that gearing should only occur following the active application of an energy input, and be completely halted in its absence. While this problem can be circumvented by mounting the gears to a surface [19, 40, 41, 42], which allows them to instead be activated electronically through the tip of a scanning tunneling microscope, for the focus of this article—gearing in solution—other approaches are needed. To this end, gears with ON/OFF states have been put forth [43, 44, 45, 46]. For these, depending on the experimental conditions, the ON state allows gearing, whereas the OFF state does not. More precisely, there are two different types of OFF states. In the first one, gearing is prevented by simply separating the two wheels of the gear from one another. In practice, this can be done by chemically modifying the backbone structure supporting the two wheels, as effectuated through the reversible attachment of a fluoride ion [43], a change in oxidation state [44], or the reversible complexation with a metal ion [45]. For the second type of OFF states, any kind of wheel rotation is prevented by the controllable intercalation of a bulky functional group between the cogs of the wheel. Typically, systems with this characteristic are referred to as molecular brakes [46], and while they have not been combined with proper molecular gears featuring two or more wheels, their ability to allow or deny free rotation of propeller‐shaped molecules (triptycene and others) is well documented [47, 48, 49, 50, 51, 52, 53].

**FIGURE 4:**
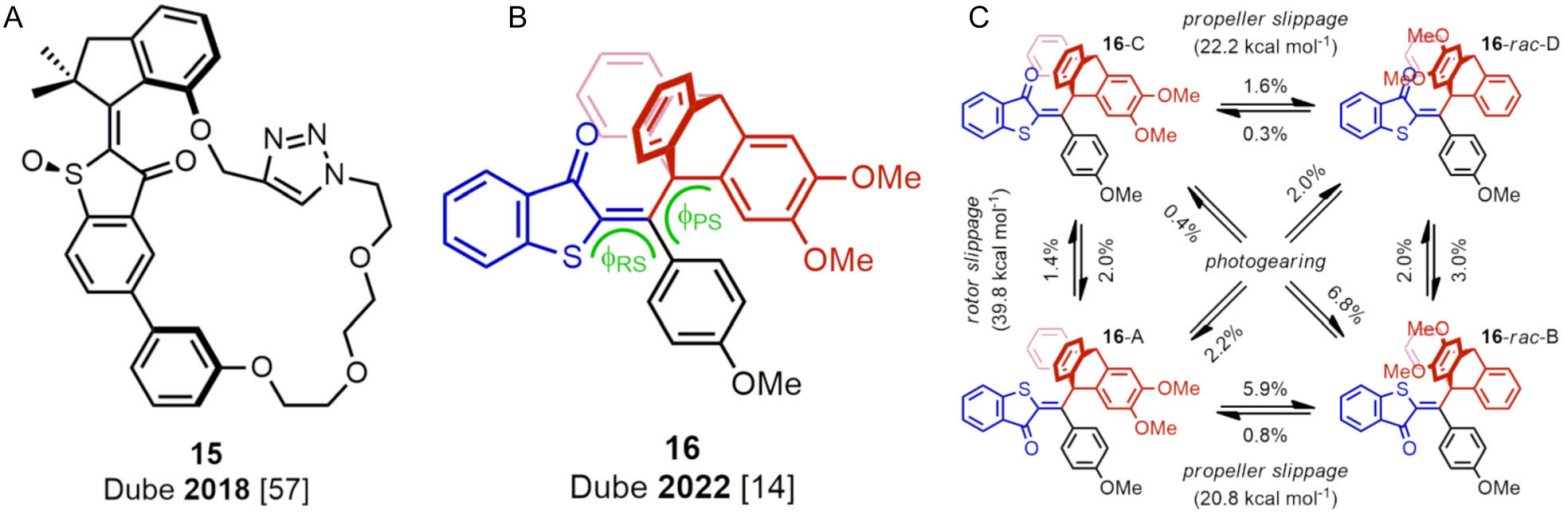
(A) Molecular motor developed in Ref. [57] and whose rotary motion is transferred to a remote biphenyl axis through an ethylene glycol chain. (B) Photogear developed in Ref. [14] with dihedral angles describing rotor (ϕ_RS_) and propeller (ϕ_PS_) rotations, and with the rotor, propeller and stator parts blue‐, red‐, and black‐colored, respectively. (C) Isomerization matrix for the photogear developed in Ref. [14] with quantum yields for photochemical processes given as percentages, and with free‐energy barriers for thermal processes given in kcal mol^–1^.

While all of these efforts have produced many insights, it is still the case that any thermally activated, solution‐based molecular gear will inevitably be sensitive to random thermal fluctuations (Brownian motion). Accordingly, the ON state of any such gear is likely to show spontaneous (uninduced), non‐directional rotation, which is not what one wants for a nanomachine. Pleasingly, however, a possible solution to this problem is to exploit the highly controllable conversion of light energy into directional rotary motion around a double bond offered by light‐driven molecular motors [54]. Along those lines, in 2017, Feringa and coworkers showed that such a motor can indeed be made to control the rotation of a naphthalene propeller around a CC single bond, although the resulting system prevented, rather than promoted, the rotation [55]. Later, in 2018 and 2020, Dube and coworkers presented two light‐driven motors of hemithioindigo (HTI) type [56] featuring a customary photoisomerizable central CC double bond. However, additionally, the motors were covalently linked to a remote biphenyl via an ethylene glycol chain, as shown in Figure 4A (structure **15**) [57, 58]. While this chain prevents, mechanically, free thermal rotation around the CC single bond constituting the biphenyl axis, activating the motors under irradiation creates a torque sufficiently high to pull the chain and transmit the torque to the biphenyl, causing its controlled rotation.

Then, in 2022, the first light‐activated molecular gear capable of transmitting rotary motion through spatial interactions alone (the hallmark of true molecular gearing [20]), without the help of a covalent chain linkage, was reported by the Dube group, who introduced the term “molecular photogear” for such a system [14]. The corresponding bevel‐like structure, shown in Figure 4B (structure **16**), is similar to the original gears in Figure 1 [12, 13, 20], but has one of the triptycene wheels replaced by an HTI photoswitch. In this structure, three separate components can be distinguished: the mobile rotor (blue‐colored in Figure 4B) that undergoes a double‐bond rotation induced by light; the mobile propeller (red‐colored) that undergoes a single‐bond rotation induced by the rotor; and the immobile stator backbone (black‐colored) that holds both the rotor and the stator (since all three components share a common carbon center, the stator is perhaps less easily perceivable).

In order to understand how the photogear in Figure 4B functions, a useful starting point is to consider its relaxed OFF state, in which the rotor resides between two of the phenyl cogs of the propeller, thus ensuring that undesirable propeller slippage (free‐standing thermal propeller rotation) is obstructed. Specifically, the Dube group showed that temperatures of ~80°C are needed to trigger propeller slippage, and even temperatures of ~120°C to trigger rotor slippage (free‐standing thermal rotor rotation) [14]. Accordingly, as indicated in Figure 4C, these experiments predicted large free‐energy barriers of ~20 and ~40 kcal mol^–1^ for these processes, respectively [14]. Following light absorption, the photogear subsequently enters a transient ON state, in which the rotor rotation induced by the ensuing CC double‐bond photoisomerization couples to a simultaneous rotation of the propeller. In other words, light absorption triggers a proper gearing process, which continues until the initial OFF state is recovered (following internal conversion from the excited state to the ground state). Carrying out the light‐irradiation experiments in isomer‐enriched solutions, the Dube group found that photogearing can occur in any of four different isomers, although, as indicated in Figure 4C, it is particularly efficient in one of them, which attains a photogearing quantum yield of ~7% [14]. Moreover, despite the absence of a permanent source of chiral asymmetry in the photogear, in a follow‐up study the gearing was found to occur with a rather pronounced directional bias, albeit with no overall directionality [15].

## 4 Directional Molecular Photogears

The reason why it is desirable for the coupled rotation achieved by a molecular photogear to exhibit an overall directionality, is that the photogear will then—over the course of several reaction cycles—be able to perform actual mechanical work (i.e., mechanical *net* work). Indeed, without this property, clockwise and counterclockwise rotations will be equally probable, prohibiting net work from being produced and limiting functionality. In this light, the development of directional molecular photogears is very much an ongoing endeavor, to which we have begun contributing [16, 17]. In particular, in our work, we have used a computational approach, combining static quantum mechanical (QM) calculations and non‐adiabatic molecular dynamics (NAMD) simulations [59]. Such an approach comes with several advantages. For example, compared to experimental studies, it allows for faster and cheaper screening of candidate photogears. Furthermore, as can be inferred from several NAMD‐based studies of light‐driven molecular motors [60, 61, 62, 63, 64, 65, 66, 67, 68, 69], it enables monitoring, understanding and tailoring the nuclear motion shown by candidate photogears along their reaction cycles, as well as calculating key quantities like excited‐state lifetimes and photoisomerization quantum yields.

Of course, the computational approach is also not free from limitations, as the accuracy of any prediction will only be as good as the chosen level of theory, with the best levels still restricted to relatively small systems. Needless to say, one also needs to infuse the modeling with some knowledge of organic chemistry, or else one cannot assess the synthetic feasibility of any tentative photogear structure emanating from the modeling. Moreover, NAMD simulations come with challenges regarding the timescales that can be modeled and the treatment of environmental (e.g., solvent) effects. As for the former, first‐principles simulations [59] based on density functional or correlated molecular orbital theory (which are often needed for accurate predictions) are usually too expensive for processes extending beyond the ps regime, although this problem can be alleviated by simulations based on semiempirical methods [70, 71]. As for the latter, while hybrid QM/molecular mechanics (MM) techniques enable the inclusion of environmental effects in NAMD simulations at the level of MM [68, 71], this approach is not yet sufficiently streamlined for screening large sets of candidate photogears.

Our own work in the field started from the basic observation that light‐driven molecular motors owe their characteristic ability to produce directional rotary motion around a double bond to a combination of steric effects and the presence of at least one stereogenic center, which break the planar symmetry of the rotor‐stator core and favor rotation in one specific direction over the other [54]. With this in mind, we decided to explore the possibility to design a directional photogear by incorporating chiral asymmetry into a multicomponent overcrowded‐alkene structure (overcrowded alkenes form a main class of light‐driven molecular motors [54]). In principle, there are three possibilities, depending on which component(s) of the photogear the asymmetry‐inducing group(s) is (are) attached: the stator, the rotor, and/or the propeller. Together, the rotor and the propeller constitute the two wheels of the photogear.

**FIGURE 5:**
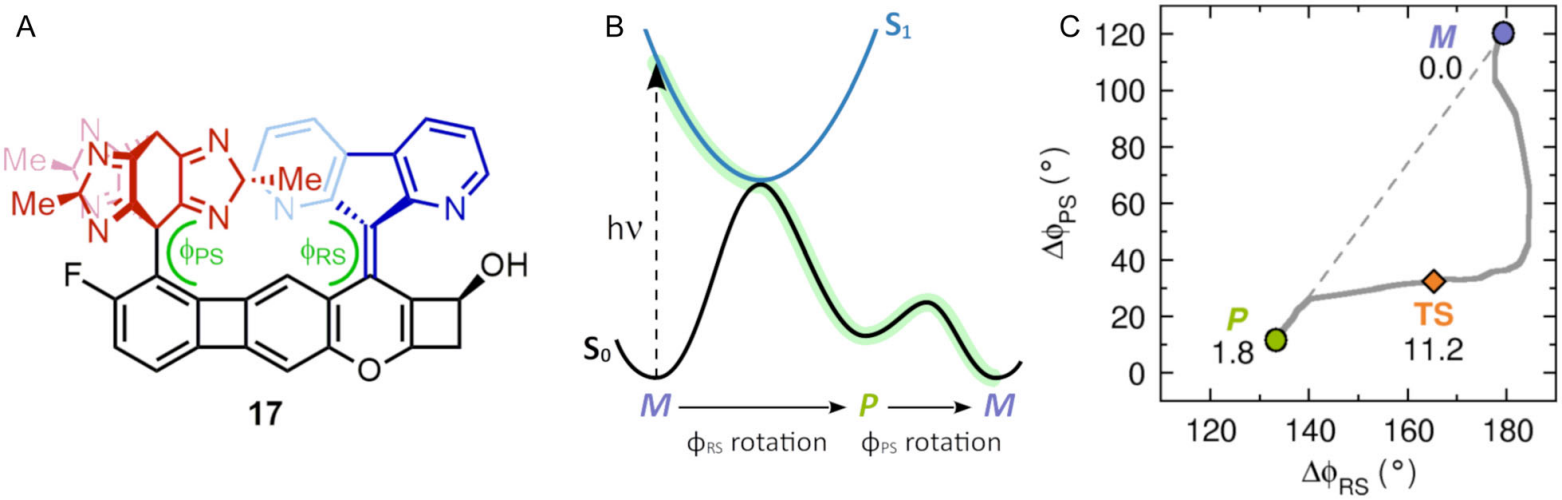
(A) Photogear developed in Ref. [16] with dihedral angles according to Figure 4B. (B) Schematic illustration of how the rotor rotation occurs in the excited S_1_ state and the propeller rotation in the ground S_0_ state. (C) Gearing during the thermal *P*/*M* helix inversion and the associated changes in the ϕ_RS_ and ϕ_PS_ dihedral angles as predicted by QM calculations. Shown are also the relative QM energies (in kcal mol^–1^) of the *P* and *M* isomers and the transition state (TS) connecting them [16]. Importantly, the corresponding ~9 kcal mol^–1^ gearing barrier was found to be ~3 kcal mol^–1^ smaller than the barrier for propeller slippage [16].

The spur photogear design resulting from this endeavor is shown in Figure 5A (structure **17**) [16]. Combining a C_2_‐symmetric diazafluorene rotor and a C_3_‐symmetric tris‐imidazole‐like propeller linked to an extended stator, this “proof‐of‐principle” design has three stereogenic centers on the rotor and another on the stator. Pleasingly, as confirmed by both QM calculations and MD simulations, the design follows a directional photogearing cycle with alternating CC double‐bond photoisomerization and thermal *P*/*M* helix inversion steps [16]. Furthermore, while Figure 5B,C show that gearing (i.e., transfer of rotation from the rotor to the propeller) occurs mostly during the thermal step, the gearing process is triggered by the preceding photoisomerization. Thus, it is appropriate to refer to this system as a photogear. Specifically, the photoisomerization of the *M* helix into the *P* helix enables gearing starting from the *P* helix by doubling the barrier of the competing propeller slippage process for the *P* helix relative to its value for the *M* helix [16].

Although this work helped unveil the untapped potential of a computational approach to the design of directional photogears, the exact structure in Figure 5A would be difficult to synthesize and is therefore of limited practical utility. Chiefly, this is because of the complexity of the stator, which includes functional groups to control the relative stabilities of the *M* and *P* isomers, which are the only two isomers possible for this photogear. Moreover, in order to optimize the distance between the two rotation axes, the stator combines aromatic and nonaromatic rings of different sizes.

Against this background, we then decided to try to design a photogear that would be easy to synthesize and whose reaction cycle would be maximally simple [17]. To this end, we changed to a bevel design, for which it is less critical that the distance between the two rotation axes is optimal [34, 35, 36]. Furthermore, attempting to eliminate thermal steps from the reaction cycle, the overcrowded alkene used for the rotor‐stator core in the former design was now replaced by a protonated Schiff base, on the grounds that such a motif has previously been predicted (computationally) to enable light‐driven molecular motors to produce directional rotary motion in a purely photochemical fashion [61, 64]. In this context, it is appropriate to mention that photon‐only motors of other types have actually been synthesized and also experimentally verified [67, 72, 73]. As for the rotor part of the photogear, we chose a C_2_‐symmetric cyclopentane featuring two stereogenic centers. As for the propeller, we chose a C_3_‐symmetric barrelene (which can be thought of as a miniature triptycene with the phenyl groups replaced by double bonds).

**FIGURE 6:**
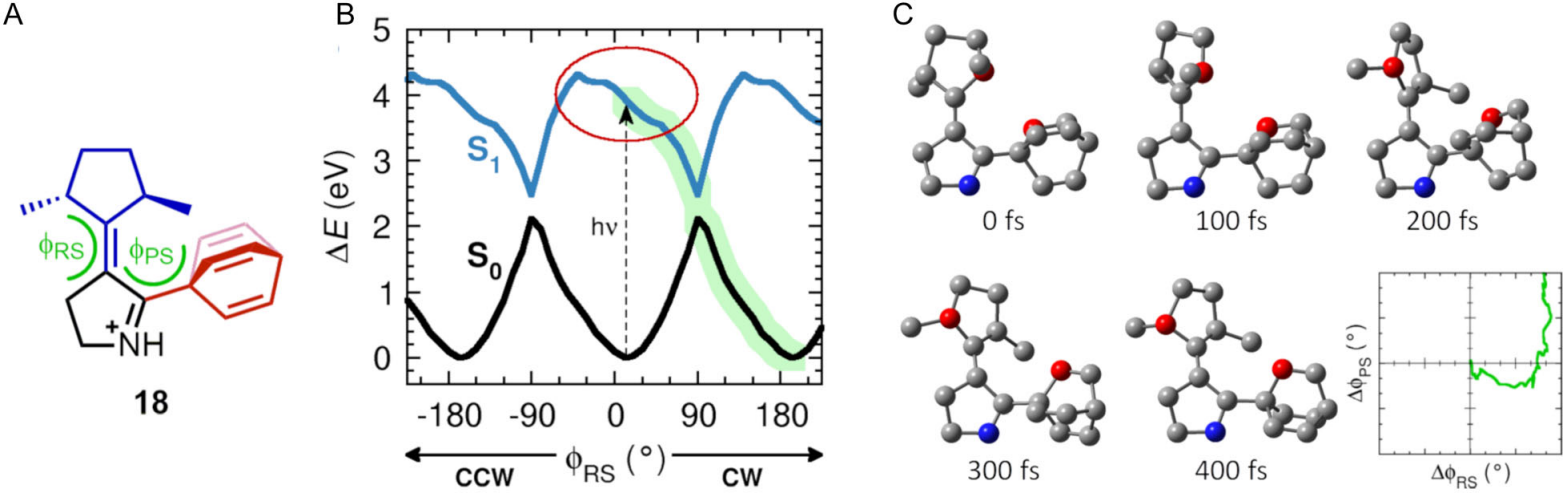
(A) Photogear developed in Ref. [17] with dihedral angles according to Figure 4B. (B) Rotor rotation preferring the clockwise (CW) direction over the counterclockwise (CCW) direction, as predicted by QM calculations [17]. (C) Propeller rotation occurring only once the rotor rotation has almost been completed, as predicted by structural snapshots at 100 fs intervals from NAMD simulations (for ease of interpretation, all H atoms are hidden and two C atoms are highlighted in red color) [17].

The resulting photogear design (whose every component is synthetically feasible [74, 75]) is shown in Figure 6A (structure **18**) [17]. Interestingly, testing the design by means of both QM calculations and NAMD simulations, it was found to allow for directional photogearing through a very simple mechanism: the reaction cycle involves just one single isomer and photogearing occurs when this isomer reforms itself in a single photochemical step [17]. Moreover, the photogearing was observed to be quite asynchronous, with the propeller rotation lagging behind the rotor rotation. Specifically, the markedly directional rotor rotation (see Figure 6B) induces a propeller rotation only when it has almost been completed (see Figure 6C). Overall, we believe that also these results provide a clear illustration of what computational modeling can bring to the field.

## 5 Summary and Outlook

Ever since the first molecular gears were reported by Iwamura and Mislow 45 years ago [12, 13], sustained efforts by numerous groups in developing new synthetic approaches, characterization techniques and computational tools have helped make this one of the most exciting fields in molecular nanoscience. However, despite the continuous emergence of new gears, with just a few selected examples given in Figures 2–6, the era has not yet arrived where gears of these types are routinely used to execute mechanical work. In order for future research to realize this goal, expanding the current plethora of thermally activated gears with photogears is a sensible path, given the latter's natural ability to carry out mechanical work (being based on directional light‐driven molecular motors).

As for key challenges along this path, we have already identified synthetic accessibility as one. Furthermore, finding experimental evidence that a photogearing process maintains a directional bias is a major task, as discussed by Dube and coworkers [15]. Another important issue (fatigue resistance [76]) is to prevent side reactions that compete with the light‐powered reactions responsible for the photogearing, and whose occurrence has a negative impact on the ability of a photogear to undergo multiple reaction cycles. Akin to how the efficiency of thermal gears is often assessed in terms of their gearing fidelity (the ratio of the rate constant or barrier for gearing to the rate constant or barrier for propeller slippage), it is also desirable to develop a similar parameter that is applicable to photogears. Here, the main issue is finding a balanced way to compare a photochemical gearing reaction to a thermal slippage process.

In our own computational work, we will next investigate to what extent the key features of the photogear in Figure 6A represent general design principles for the construction of photogears. For example, having found the photogearing of this system to be quite asynchronous, it is of interest to examine whether a higher degree of synchronicity improves the directional bias. Moreover, having employed a small barrelene propeller, it is imperative to test whether replacing this motif with its full triptycene counterpart strengthens the coupling of the two rotations. Similarly, having used rotor and propeller motifs with C_2_ and C_3_ symmetries, it is pertinent to explore how similar motifs with other symmetries perform.

## Funding

This study was supported by Vetenskapsrådet (2019‐03664, 2022‐06442), Olle Engkvists Stiftelse (204‐0183), and Carl Tryggers Stiftelse för Vetenskaplig Forskning (CTS 24:3446).

## Conflicts of Interest

The authors declare no conflicts of interest.
